# Advances in Multi-Omics Applications in HBV-Associated Hepatocellular Carcinoma

**DOI:** 10.3389/fmed.2021.754709

**Published:** 2021-09-30

**Authors:** Dawei Cui, Wei Li, Daixi Jiang, Jianguo Wu, Jue Xie, Yingping Wu

**Affiliations:** ^1^Department of Blood Transfusion, The First Affiliated Hospital, Zhejiang University School of Medicine, Hangzhou, China; ^2^Center of Research Laboratory, The First People's Hospital of Lianyungang, Lianyungang, China; ^3^State Key Laboratory for Diagnosis and Treatment of Infectious Diseases, National Clinical Research Center for Infectious Diseases, Collaborative Innovation Center for Diagnosis and Treatment of Infectious Diseases, The First Affiliated Hospital, Zhejiang University School of Medicine, Hangzhou, China; ^4^Department of Laboratory Medicine, The Fourth Affiliated Hospital, Zhejiang University School of Medicine, Yiwu, China

**Keywords:** hepatitis B virus, hepatocellular carcinoma, metabolomics, proteomics, genomics, transcriptome, non-coding RNA

## Abstract

Hepatitis B virus (HBV) specifically infects liver cells, leading to progressive liver cirrhosis and significantly increasing the risk of hepatocellular carcinoma (HCC). The maturity of sequencing technology, improvement in bioinformatics data analysis and progress of omics technologies had improved research efficiency. The occurrence and progression of HCC are affected by multisystem and multilevel pathological changes. With the application of single-omics technologies, including genomics, transcriptomics, metabolomics and proteomics in tissue and body fluid samples, and even the novel development of multi-omics analysis on a single-cell platform, HBV-associated HCC changes can be better analyzed. The review summarizes the application of single omics and combined analysis of multi-omics data in HBV-associated HCC and proposes the importance of multi-omics analysis in the type of HCC, which provide the possibility for the precise diagnosis and therapy of HBV-associated HCC.

## Introduction

Globally, 830,180 people died of cancers in 2020, and liver cancer became the third causative factor of cancer-associated death (8.3% of 9.9 million deaths) (1). Hepatocellular carcinoma (HCC) is the most dominant primary liver cancer, which can be caused by hepatitis B virus (HBV), hepatitis C virus (HCV), alcohol abuse, and so on (2, 3). Although acquired HBV infection has been well-controlled by vaccines, HBV remains the main cause of HCC due to nearly 300 million individuals with chronic HBV (CHB) infection worldwide (4). It is estimated that 8–20% of untreated patients with CHB infection will progress to liver cirrhosis within 5 years (5), and ~2–8% of the patients with liver cirrhosis can be transformed into HCC (6). Persistent HBV infection or active HBV replication results in liver injury, fibrosis, cirrhosis, and liver cancer, leading to most of the end-stage liver diseases (7, 8). Up to one-third of patients with HBV-associated HCC will develop cirrhotic tumors (2). Additionally, inactive HBV carriers with serum alanine aminotransferase (ALT) levels in the normal range have substantial risk of HCC compared to those without HBV infection (9).

Nucleos(t)ide analogs (NAs) and PEG-interferon are recommended antiviral treatments in routine medicine (such as lamivudine, adefovir, dipivoxil, entecavir, and tenofovir) that can prevent viral replication and CHB progression. However, these drugs don't affect the HBV genome in the host liver cells, which has always been in the form of covalently closed circular DNA (cccDNA) (10). Additionally, HBV-associated HCC presents more chemoresistance than non-HBV tumors. Thus, current treatment regimens are not curative, and the primary objective of therapy for CHB infection is to permanently inhibit HBV replication, followed by lifelong therapy (11). Given the above limits in treatment and huge scale of HBV infection worldwide, new therapeutic strategies are necessary.

CHB infection is dynamic interactions among the hepatocytes, virus and immune system of the host. In recent years, significant progress has been made in genome and proteomic analysis, clinical data management, next generation sequencing data mining, machine learning and deep learning algorithms. “Omics” technologies are used to mainly detect all protein, transcripts, and metabolites for mining for available data in the biological sample. These high-throughput technologies play critical roles in describing gene and/or protein expression profiles, and their effects on HBV-associated HCC (12–14). Although many biomarkers for diagnosis and prognosis have been identified through omics analysis of HBV infection, the previous studies focus on a single aspect of the natural history of CHB. However, most studies on systematic omics are based on genomics, transcriptomics and proteomics. The integration of multi-omics data analysis is critical for providing novel insights into the transitions and molecular mechanisms in related diseases (15, 16).

With the technological advances of platforms, multi-omics analysis will be more crucial for molecular therapies and precision medicine. Integrative analysis of multi-omics platforms mainly relies on innovative technology platforms including genomics, metabolomics, and proteomics. Multi-omics studies have been successfully exploited to elucidate the pathogenic mechanism of infectious diseases, such as helicobacter pylori (HP)-associated gastric carcinoma (17), COVID-19 (18), and herpes simplex virus-1 (HSV-1) infection (19). The promise of the multi-omics approach has been well-described in more complex diseases, and several studies have proposed potential biomarkers for HCC using omics resources. Although “omics-level” studies have been very useful in understanding the mechanism of HCC manifestation, few are available to integrate different omics data.

**Figure 1:**
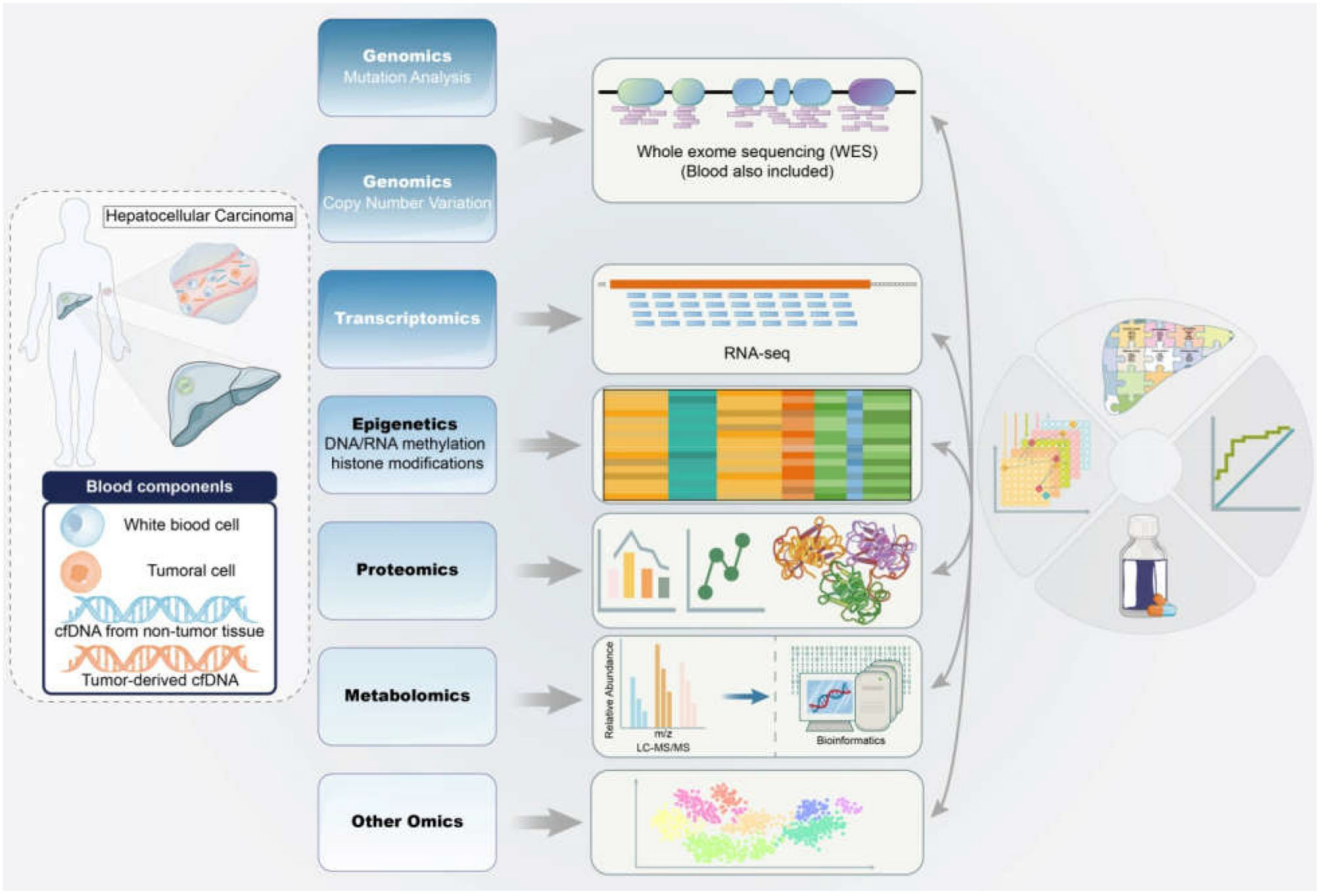
Application of multi-omics in the diagnosis and therapy of HBV-associated HCC. The samples from HBV-associated HCC subjects were detected and analyzed by mitiomics technology including genomics, transcriptomics, epigenetics, proteomics, metabolomics and other omics to accurately promote biomarker-driven treatments and immunotherapies for HCC patients. HCC, hepatocellular carcinoma; HBV, hepatitis B virus; RNA-seq, RNA sequencing; LC-MS/MS, liquid chromatograph-mass spectrometer/mass spectrometer.

Herein, we review recent advances in multi-omics applications, including genomics, epigenetics, transcriptomics, proteomics, and metabolomics. Overall, this review will highlight the omics advances in HBV-associated HCC to provide novel insights into immunotherapies based on specific biomarkers in the future (Figure 1).

## Epidemiology

Hepatitis B virus is a DNA virus with a partial double-stranded relaxed circular DNA genome, containing four open reading frames (ORFs) with *P, pre-S/S, pre-C/C*, and *X* genes, and *Pre-S/S* comprises the *pre-S1, pre-S2*, and *S* genes. The *P* region encodes DNA polymerase and RNase H, which is associated with virus replication. *Pre-C/C* encodes hepatitis B core antigen (HBcAg) and hepatitis B e antigen (HBeAg), and *X* region encodes a hepatitis B x (HBx) non-structural protein, which is involved in viral replication and oncogenic activity. A recent report showed that the HBx protein could promote the degradation of the structural maintenance of chromosome (SMC) 5/6 protein complexes to increase HBV replication and indirectly proves the association with the occurrence of HCC again (20).

Mutations of the viral genome causing biological behavior changes may have crucial effects on HBV pathogenicity and are closely associated with the malignant transition of liver cancer (21). The occurrence of HBV-associated HCC is a complex process. HBV infection can promote HCC through direct or indirect mechanisms, including HBV gene integration, genomic instability, and activation of cancer-associated signaling pathways. Additionally, new insights into the mechanism of HCC-related pathway activation, including epigenetics, autophagy, exosomes, metabolism, and immune responses (22–25), are being continuously focused on. Previous studies also showed that HBV-associated HCC individuals displayed distinctive profiles including chromosomal alterations and β-catenin mutations (26). Furthermore, genetic alterations in subgroups of HCC cases were remarkably associated with HBV-DNA levels (27). Additionally, novel biomarkers, such as DNA mutations, DNA or RNA methylation, long non-coding RNAs (lncRNAs), microRNAs (miRNAs), and circular RNAs (circRNAs), are under investigation and can be considered for future clinical practice of HCC.

Thus, integrated multi-omics analysis must be performed to obtain a better understanding of the pathogenesis of HBV-associated HCC. The combined application of genomics, epigenomics, and transcriptomics to illustrate the mechanism of virus-associated carcinogenesis is required.

## Genomics Characteristics

In contrast to HCV, HBV can integrate the viral genome into the host hepatocyte genome. A thorough understanding of the pathogenesis of virus-associated carcinogenesis is critical for early diagnosis, treatment and prevention of HCC. Recent advances in deep sequencing technologies including next-generation sequencing, nanopore sequencing, and single-cell sequencing, contribute to revealing the landscape of genetic and epigenetic changes in tumor tissues and chronic liver damage caused by hepatitis virus infection, particularly HBV (28). Persistent suppression and/or eradication of HBV/HCV can contribute to reducing the incidence of HCC, but multi-centric tumors in patients often arise after viral clearance (29–31). Additionally, the accumulation of viral genetic alterations negatively affects the epigenetic transformation of normal cells into cancer cells (32). NGS technologies, including whole-exome sequencing (WXS), RNA sequencing (RNA-seq), and whole-genome sequencing (WGS), form the basis of current genomics research. Therefore, vast amounts of sequencing data have been shared in global databases, allowing researchers to synthesize analyses that lead to new findings.

### Host Profile

Genetic aberrations comprise nucleotide changes and structural variations (STVs) (33–35). The accumulation of somatic genomic alterations in primary tissues is the major cause of HCC. An average of 40–60 somatic alterations is detected in the protein coding regions of the genomes from HCC patients (36) (Table 1). Based on WES and single-nucleotide polymorphism (SNP) array analysis, how these mutated genes and the copy number of their alterations are involved in regulating these pivotal pathways, including cell cycle control, telomere maintenance, chromatin modification, and receptor tyrosine kinase, which have been reported (51). Among these mutations, a few genomic alterations are considered to be directly involved in the activation of the important signaling pathways for hepatocarcinogenesis.

**Table 1:** The common somatic genomic alterations and HBV integration events in HCC.

**Genomic aberration**	**Aberration frequency (% of patients)**	**HBV integration events**	**Pathway**	**Biological roles**	**References**
*TP53*	3–40% (mutations); 2–15% (loss)	No	P53 pathway	Tumor suppressor	(37, 38)
*CTNNB1*	11–41%	No	Wnt pathway	Regulation in cell adhesion, growth, and differentiation	(38, 39)
*ARID1A*	5–15%	No	Chromatin remoedling	Transcriptional activation of selective genes and inhibition of chromatin remodeling	(39)
*ARID2*	3–15%	No	Chromatin remoedling	Tumor suppressor gene in the transcriptional activation and inhibition of specific genes	(40, 41)
*JAK1*	7.70%	No	JAK1/STAT3 pathway	Good prognostic marker for survival of HCC patients	(25)
*AXIN1*	5–19%	No	Wnt pathway	As signal transducer to regulate cell adhesion, growth, and differentiation.	(37, 42)
*CDKN2A*	7–8%	No	cell cycle	Tumor suppressor genes that promote cell cycle arrest in G1 and G2.	(39)
*KEAP1*	2–8%	No	Oxidative stress pathway	Proteinase adaptor	(14)
*ARID2*	3–15%	No	Chromatin remodeling	Growth hormone receptor	(40, 41)
*FGF family members (FGF3, FGF4, FGF19)*	4–5.6%	No	FGF pathway	Mitogenic and cell survival activities	(36)
*TERT*	~60%	Polymerase, X protein, Precore/core protein	Telomere maintenance	Telomere repeat (TTAGGG) was added to the end of chromosome;the erosion of telomere protective end was compensated.	(39, 40, 43, 44)
*MLL4*	3%	Polymerase, X protein	Chromatin regulators	Epigenetic modification	(45, 46)
*CCNE1*	10%	X protein, Precore/core protein, S	TP53 /cell-cycle pathway	Strong CcnE1 overexpression is correlating with poor prognosis of HCC patients.	(47, 48)
*FN1*	No	Precore/core protein, X protein, polymerase	No	FN1 promotes the migratory and invasive of hepatoma cells.	(40, 45, 47, 49)
*CDK15*	No	S, Polymerase, X protein, Precore/core protein	No	Protein Coding gene	(41)
*ROCK1*	No	X protein, S	No	Stable overexpression of ROCK2 remarkably promoted cell motility and invasiveness in HCC cells.	(50)
*ApoA2*	No	Polymerase, X protein, S	No	Apolipoprotein family	(50)

The integration of viral genome is a unique molecular characteristic of HCC. Notably, the integration of HBV genome affects gene expression near integration sites. Multiple recurrent genetic aberrations and the disruption of the host genome due to HBV-DNA integration are important for the hallmarks of HBV-associated HCC. By large-scale genome sequencing analysis of HCC, the core drivers (*TERT, TP53*, and *CTNNB1/AXIN1*) have been identified as initial molecular events and other low-frequency drivers including therapeutically targetable drivers. These genes regulate some pathways, including cell cycle (*p53, p16*), apoptosis (*bcl2*), cell proliferation and differentiation (*b-catenin, c-myc, APC, E-cadherin*), metastasis (*MMP4, MMP9, Topoisomerase, Rb, Cyclin D1, Osteopontin*), angiogenesis (*VEGFR-2, Angiopoietin-2*), and other growth factor signaling components (*IGF-II, TGF, EGFR, HGF/c-MET, PTEN, K-RAS)* (37, 42). These findings indicate that HCC is not caused by a specific driver mutation but involves in the multiple carcinogenic pathways that enhances extremely heterogeneous of HCC.

By next-generation sequencing, somatic mutations in *TP53, TERT* promoter, and *CTNNB1* have often been reported in HCC patients (38). Somatic mutations are abundant in *TERT* gene promoters and occur in more than 50% of the patients with HCC, while the protein alterations caused by gene mutations are often observed in *CTNNB1* genes and *TP53*. Additionally, somatic structure variants (SVs) affect gene expression in cancers. The Pan-Cancer Analysis of Whole Genomes (PCAWG) Consortium revealed that 100-kb SV breakpoints for hundreds of genes were associated with their altered expressions by aggregating whole-genome sequencing data from a cohort of 1,220 cancer individuals. For most of these genes, SVs result in increased expression rather than decreased expression, and the up-regulated cancer-related genes included *TERT, CDK4, MDM2, ERBB2, PDCD1LG2*, and *IGF2* (39). Simultaneously, WGS analysis demonstrated several important types of SVs in the genome of liver cancer, including *TERT, APC, CDKN2A, ARID1A*, and new genes such as *TTC28, LRP1B*, and *MACROD2*, and these SVs affected their expressions (39).

The increased copy number of HBV-DNA at HBV breakpoint locations indicates that chromosomal instability is associated with HBV genome integration (52). Hama et al. identified the structural rearrangement that integrated the viral genome by WGS analysis in HBV-associated HCC (50). Therefore, the structural instability of the integrative viral genome is periodic and may be related to the chromosomal instability of the host hepatocyte genome.

### HBV Profile

Approximate 350–400 million people worldwide are infected by HBV, and persistent HBV infection leads to more than 50% of HCC patients. HBV plays an important role in the development of HCC by integrating the HBV genome into the host genome. Several high-throughput sequencing studies have reported that HBV genome integration occurs in a high rate of HBV-associated HCC patients (Table 1) (52). Although HBV can randomly and repeatedly integrate into the host genome including *TERT and MLL4*, suggesting functional consequences for the host by HBV integration events (45). Some studies have also identified that the region between 1,600 and 1,900 nucleotides within the viral genome, corresponding to the 3′-end of HBx gene and 5′-end of precore gene, is not only preferentially involved in structural alterations within the viral genome, particularly deletion and inversion events, but also significantly related with the insertion into the host genome (43, 53). In a previous study, HBV genome integration was significantly enriched on the q-arm of chromosome-10 in a cohort of 48 HCC cases, and the event was related with poorly differentiated tumors (43). In several studies, HBV has been reported to integrate into the *CCNE1* and *TERT* genes. However, *CDK15, ROCK1, FN1, ApoA2*, and *MLL4* have rarely been reported as HBV integration sites (40, 47, 49). In 76 samples of HBV-associated HCC cases, 4 cases of HBV integration within *CCNE1* were reported, resulting in the high expression of *CCNE1* (47). Multiple high-throughput genomic studies have found that repeated integration sites on *TERT* promoters are the most frequent integration sites (40). Disruption of the *telomerase reverse transcriptase (TERT)* promoter may result in the dysregulation of *TERT* expression (44). The mRNA expression of *TERT* is increased when HBV binds to the *TERT* transcription start site, which implies HBV sequences as enhancers for *TERT* mRNA expression (43). In a Chinese cohort of forty-four HBV-associated HCC tissues, 8 fusion transcripts of *HBx/MLL* were found, leading to high expression of the *MLL4* gene (46). Furthermore, multiple transcripts with HBV-CDK15 fusion were observed in an HCC case, including one in-frame fusion, which induced CDK15 overexpression (41). Hence, the genes of *CCNE1, ANGPT1*, and *TERT* are not only mutated in somatic cells, but also integrated in viruses (48).

## Epigenetics Characteristics

Viruses can alter the chromatin structure by redirecting the modifications of chromatin and consequently affecting host cell transcription, which may contribute to oncogenesis (54, 55). Epigenetics refers to changes of gene expression without altering the underlying DNA sequence, and comprises three major components: histone modifications, DNA methylation, and non-coding RNA mechanisms (56). Hepatocellular carcinoma is caused by the somatic mutations leading to the abnormalities of chromatin regulations and epigenetic characteristics (57). In the cases with HBV infection, the disorders of DNA/RNA methylation and histone modifications have been reported, but which have focused on specific genes or pathways, and genome-wide mapping of the epigenetic alterations is rare.

### DNA Methylation

DNA methylation in many tumor suppressor genes is related with carcinogenesis. Through an array-based platform, the genomic DNA methylation pattern of nearly 200 patients with HCC further revealed the different cancer-specific DNA hypermethylation clusters (58). DNA methylation is significantly different among HCV-related HCC, HBV-associated HCC and normal tissue (Table 2) (66). However, some evidences support more prominent DNA methylation alterations in HCV-associated HCC than in HBV-associated HCC (67). Similar to other cancers, HCC is characterized by the global DNA hypomethylation and promoter hypermethylation, which are related with the up-regulated tumor-promoting genes (68, 69). High frequencies of aberrant DNA hypermethylation of specific genes (*GSTP1, RASSF1A, DOK1*, and *CHRNA3*) in HCC were reported, and these genes was suggested as a prognostic marker of HCC combined with clinicopathological data (59, 60). Furthermore, a recurrent hypomethylated enhancer of CCAAT/enhancer-binding protein-beta (C/EBP-β) promoted HCC tumorigenicity through global transcriptional reprogramming (70). Methylation of the *APC, RASSF1A*, and *GSTP-1* genes is associated with HCC (61–64). Apart from methylation at gene promoters and CpG islands, epigenetic regulation and genome-wide enhancer hypomethylation patterns in primary human HCCs must be elucidated by whole-genome sequencing. In HCC patients, the latest three reports identified 6 CpG sites in white blood cell (WBC) DNA and showed that DNA methylation at those sites could distinguish HCC from healthy blood in prospective samples taken before diagnosis (71–73). Additionally, compared with hepatitis and cirrhosis liver tissues, increased DNA methylation of CpG island 3 in the HBV genome indicated HBV methylation in HBV-associated HCC pathogenesis (74). Furthermore, HBV infection induces gene methylation in HCC (75). HBV infection promotes the activity of DNA methyltransferase, which causes the simultaneous methylation of host CpG islands and HBV-DNA in cell experiments (76).

**Table 2:** DNA methylation in HBV-associated HCC.

**DNA methylation**	**Methylation status**	**Biological functions in HCC**	**References**
RASSF1A	Hypermethylation	As a diagnostic and prognostic non-invasive biomarker for HCC.	(59, 60)
*GSTP1*	Hypermethylation	As a diagnostic marker, GSTP1 methylation can obviously enhance the risk of HBV-associated HCC patients with cirrhosis.	(59–61)
*DOK1*	Hypermethylation	A tumor suppressor gene, and methylation level of *DOK1* is inversely related with gene expression.	(59, 60)
*APC*	Hypermethylation	Methylation of *APC* could involve in early stages of HBV-related HCC, coupled with RASSF1A.	(61–65)
*p16*	Hypermethylation	As a diagnostic marker, P16 methylation in promoter region could obviously increase the risk of HBV-associated HCC in patients with cirrhosis.	(37)
*MGMT*	Hypomethylation	Loss of methyl-cytosine at the MGMT gene promoter may be considered as an early and transient biomarker of hepatocarcinogenesis.	(60)

However, the potential for these markers to be used for clinical application is low because biopsies are unsuitable for early diagnosis. Because blood contains circulating tumor DNA, blood may be a promising material for carrying the same DNA methylation signals of markers as tumor tissue (71, 77). With the important role of “liquid biopsy” in identifying specific molecular signals in nucleic acids released by cancer cells, some studies have found that by detecting the methylation level of specific sites of circulating tumor DNA(ctDNA) in a small amount (4–5 ml) of peripheral blood, it can be used to accurately diagnose HCC early and to predict the curative effect and prognosis (78).

### RNA Methylation

N6-methyladenosine (M6A) is present in most eukaryotic messenger RNAs (mRNAs) and is the most commonly modified form of mammalian RNA. Recently, some studies have reported that hepatocarcinogenesis is closely related with abnormal m6A modifications (79, 80). They found that high expression of the m6A methylase METTL3 in HCC patients leads to high levels of m6A in SOCS2 mRNA, resulting in the rapid degradation of SOCS2 and HCC occurrence (79), while METTL14 had no significant effect on HCC, and down-regulation of METTL14 expression was related with a poor prognosis in HCC patients without recurrence (80). Although the relationship between DNA and RNA m6A remains unclear, at least one independent way can verify the m6A modification sites predicted by big data (81). By transcriptome sequencing, some genes related with m6A in HCC, particularly METTL3 and YTHDF2, had been confirmed to be a risk signature (79). The processing of miR-126 maturation is mediated by the methylation transferase METTL14 in HCC, and the reduced expression of miR-126 maturation will cause HCC metastasis (80). Thus, regulators of m6A modification can become potential biomarkers for prognosis in HBV-associated HCC.

### Histone Modifications

In the hepatocyte nucleus, HBV-cccDNA assembles with the histone proteins of host cells to form minichromatin, which is dynamically regulated through histone post-translational modifications (PTMs) to promote the expression of viral genes. Previous reports have revealed a series of histone modifications on HBV cccDNA, such as H3K4me2, H3K4me3, H3K9ac, H3K27ac, H3K36me3, and H3K9me3. H3K4me2, H3K4me3, H3K9ac, H3K27ac, and K3K36me3 are associated with activating gene expression, while H3K9me3 is related to gene silencing (82). A highly sensitive technique, 3C-high-throughput genome-wide translocation sequencing (3C-HTGTS), was used to identify the interactions of HBV-DNA and host DNA in which H3K4me1 histone modification is enriched by kmt2c/d, while H3K4me1 histone modification contributes to activate the transcriptional activity of HBV. They found that histone modifications not only strongly affected HBV transcription on minichromosomes of HBV cccDNA but also affected host gene expression (83). Recently, Alvarez-Astudillo et al. found that the histone variant H3.3 was assembled from the histone chaperone HIRA to the HBV-cccDNA, and this assembly was correlated with increased levels of the active H3K4me and activation of HBV transcription (84). The first genome-wide maps of PTMs obtained by chromatin immunoprecipitation sequencing (ChIP-Seq) have revealed that high levels of PTMs associated with transcription activation are enriched at specific sites in the HBV genome, whereas very low levels of PTMs are related with transcriptional inhibition, even at silent HBV promoters (82). Herein, the effect of transcriptional and active PTMs may open the possibility of chromatin regulating HBV-cccDNA transcription, providing a new way to treat chronic hepatitis B virus infection.

## Transcriptomics Characteristics

### Host-HBV Transcription

Hepatitis B virus plays a crucial role in HCC progression by integrating the viral genome into the host genome, and genome integration events are observed in HBV-associated HCC patients using high-throughput sequencing (43, 47, 52, 85). Pregenomic RNA (pgRNA) of 3.5 kb, an RNA intermediate, is critical for HBV replication. Thus, the level of HBV replication in tumors or adjacent non-tumors is assessed by the presence of intact pgRNAs in liver tissue, but few intact pgRNAs are observed, particularly in the tumor tissues of HCC patients.

Most somatic mutations in HCC are in the coding regions with potential functional effects. Five thousand four hundred and eleven tumor-specific mutations were identified with an average of 230 somatic mutations in each HCC patient, and these somatic mutations were significantly different in the distribution of the different genomic regions and their predicted functions. Moreover, deep transcriptome sequencing of HCC patients provides information on RNA expression, transcriptional mutations and characteristics of HBV-human chimeric transcripts (45). The integration characteristics of HBV were identified by RNA sequencing, and the preferred integration sites near the telomeres were reported (52). The integration sites in the structural changes within HBV and host genome have been well-described at the genomic level, but the status of HBV transcripts in HBV-associated HCC has not been comprehensively analyzed (86). To Target different HBV transcripts in depth, Stadelmayer et al. have developed an HBV full-length 5′RACE (rapid amplification of cDNA ends) method, which significantly contributes to the understanding of HBV transcription and may guide the development of new therapies targeting HBV-cccDNA (87).

HBV fusion sequences are significantly enriched on chromosome 10 (43). More HBV-human fusions (161 fusions) in non-tumorous tissues were observed in the HCC transcriptome of 22 HCC patients than in matched HCC tissues (33 fusions) (41). Notably, the data obtained through transcriptome analysis showed that most chimeric transcripts in tumors fuse with gene sequences more than at the genomic level, HBV was fused with the repetitive sequences, particularly the LINE and SINE families of the repetitive sequences in 40% of the chimeric transcripts (43).

Although transcriptome sequencing can provide valuable insights into the characteristics of HBV-associated HCC patients, most studies have focused on host transcripts rather than viral transcripts (88–90). More than 90% of HBV-associated HCC contains transient HBV-DNA integration, which does not produce all the HBV antigens, and these transient HBV-DNA fragments encode epitopes that can be recognized by activate T cells (47, 91–93). The HBV transcriptomes of the HCC cells can be used for individualized immunotherapy with engineered T cells and as a treatment measure for a wider range of HBV-associated HCC patients (93). The transcriptome similarities and differences in CD8^+^ T cell dysfunction were explored in both chronic HBV infection and HCC patients through high-throughput RNA-seq, and the results demonstrated that CD8^+^ T cell dysfunction in the two groups shared high similar characteristics, but each had its own characteristics in specific genes and signal pathways (94).

### Non-coding RNA

Non-coding RNAs (ncRNAs) are functional RNAs that cannot encode proteins. ncRNAs make up a significant proportion of cellular RNAs, accounting for more than 90% of human RNAs. Recent reports have shown that ncRNAs play an important role in multiple cellular processes including cell proliferation, apoptosis, migration, and angiogenesis. Many tumor cells including liver cancer cells, also release specific circRNAs, microRNAs (miRNAs), long non-coding RNAs (lncRNAs), and extracellular vesicles containing proteins, lipids, RNAs and miRNAs in peripheral blood (95–98). Non-coding variants are closely linked to human cancers and are even involved in drug resistance in HCC. Herein, we focused on miRNAs, lncRNAs and circRNAs implicated in the pathogenesis of HCC (Table 3).

**Table 3:** Non-coding RNAs in HCC.

**Non-coding RNAs**		**Expression change**	**Biological function**	**References**
microRNAs	miRNA Panel (miR-122, miR-192, miR-21, miR-223, miR-26a, miR-27a and miR-801)	Up-regulation	The miRNA panel can differentiate HBV-associated HCC from healthy subjects, CHB and cirrhosis, respectively.	(99)
	miR-154	Down-regulation	As a tumor suppressor, the miR-154 expression is down-regulated in HCC.	(98, 100)
	miR-519d, miR-595, miR-939, miR-494 and miR-21	Up-regulation	miR-939, miR-595 and miR-519d could differentiate cirrhotic patients with and without HCC. Moreover, miR-519d, miR-494 and miR-21 were related with the progression of HCC.	(101)
	miR-21 and miR-10b	Up-regulation	The exosomal miR-21 and miR-10b promote cancer cell proliferation and metastasis in HCC, and may serve as prognostic markers and therapeutic targets for HCC.	(102)
	miR-15a/miR-16-1	Down-regulation	HBx transcript directly drives the down-regulation of miR-15a/miR-16-1 by the miRNA targeting sequences in the viral RNA.	(103)
	miR-204, miR-1236	Down-regulation	miR-204 and miR-1236 can inhibit HBV replication involved in two different mechanisms.	(100)
LncRNAs	H19	Up-regulation down-regulation	Promote HCC growth, inhibit migration and invasion of HCC cells.	(104, 105)
	HULC	Up-regulation	Triggering autophagy *via* stabilizing Sirt1; Promoting HCC growth	(104, 106, 107)
	HOTAIR	Up-regulation	Activating STAT3/ABCB1 pathway and promoting HCC growth	(104, 108)
	MALAT1	Up-regulation	Associated with tumor metastasis, recurrence	(103, 104)
	HEIH	Up-regulation	Associated with HBV-HCC and prognosis	(109)
	HOTTIP	Up-regulation	Associated with tumor progression and disease outcome	(110)
	MEG3	Down-regulation	Associated with methylation and Inhibit cell growth	(111)
	LncRNA-LET	Down-regulation	Reduces hepatic invasion and abdominal metastases	(112)
	SRHC	Down-regulation	Inhibit cancer proliferation	(113)
	PTENP1	Down-regulation	Suppress the tumorigenic properties of HCC cells	(114)
circRNAs	circ_104075	Up-regulation	Circ_104075 as a ceRNA can upregulate YAP expression by absorbing miR-582-3p, and may provide new insights in HCC diagnosis and therapy.	(115)
	cSMARCA5	Down-regulation	It has diagnostic value for patients with alpha-fetoprotein <200 ng/mL.	(97)
	circ_0009582, circ_0037120, circ_0140117	Up-regulation	The high sensitivity and specificity of the combination of three circRNAs and AFP could be used to distinguish HBV-infected patients with and without cancer.	(116)
	circ_KIAA1429	Up-regulation	Post-transcriptional modification of circ_KIAA1429 may also play a role in influencing translation.	(117)
	circMET	Up-regulation	Inducing development and immune tolerance of HCC by the Snail/DPP4/CXCL10 axis.	(118)
	circPTGR1	Up-regulation	Associated with the clinical stage and prognosis.	(119)
	circ_−_0051443	Down-regulation	Potential therapeutic target for HCC	(120)

### miRNAs

miRNAs are one kind of important non-coding RNAs (ncRNAs), ~22 nucleotides in length, and are highly expressed in many types of tumors associated with HCC progression or suppression (101, 102). miRNAs may act as tumor suppressor genes or oncogenes by silencing and targeting mRNAs involved in carcinogenesis. Recent studies showed that miRNA expression is more valuable than mRNA-based profiling to identify tissue types of tumor origin, and cancer treatments targeting miRNAs are currently in clinical trials as early detection markers of HCC (100, 121). In the past 2 years, many reports showed that miRNAs were closely related with hepatocarcinogenesis (116). A recent study showed that miR-154 as a tumor inhibitor could suppress cell proliferation and metastasis, and the miR-154 expression was downregulated in HCC (99). Feng et al. found that lncRNA PCNAP1 promoted HBV replication by regulating miR-154/PCNA/HBV-cccDNA signal and PCNAP1/PCNA signal, which drived the growth of both HBV-associated HCC and HBV-free HCC (98). Some studies have considered that miRNA expressions play an important in the pathogenesis of HCC by the downregulation of miRNAs that upregulate oncogenes or the upregulated miRNAs that target tumor suppressor genes (122). In the “Expert consensus on early screening strategy for liver cancer in China,” serum miRNAs as a potential diagnostic marker have made some progress. In a study of 934 subjects, including groups of healthy people, patients with CHB, liver cirrhosis and HBV-associated liver cancer, 723 miRNAs were screened on a large scale in plasma samples. The results showed that seven specific miRNAs were selected to construct the diagnosis model of HCC and can distinguish liver cancer from healthy people (sensitivity for 83.3%, specificity for 93.9%), liver cancer from hepatitis (sensitivity for 79.1%, specificity for 76.4%), and liver cancer from liver cirrhosis (sensitivity for 75.0%, specificity for 91.1%) (123). At the same time, other studies have also proven that miRNAs have important value in the diagnosis of HCC, but the sensitivity and specificity of this technology must be further improved. Furthermore, the application values of miRNAs require large-scale samples and multicenter clinical verification, which can be used as a supplement for individualized diagnosis.

### LncRNAs

Benefiting from advances in the transcriptome sequencing, lncRNAs, transcripts more than 200 bp in length without encoding proteins, play roles in different physiological and pathological processes and affect cellular functions (106, 124). To date, most of lncRNAs play important roles in regulating specific cellular processes, particularly in the expressions of protein-coding genes at the epigenetic, transcriptional and post-transcriptional levels in cancer including HCC (125). Previous studies have shown that the lncRNAs MALAT1, H19, HOTAIR (HOX transcript antisense intergene RNA), HULC, and PRNCR1 are abnormally expressed in various human cancers, particularly HCC (104). LncRNAMALAT1 induces murine HCC experimentally, H19 expression is upregulated in HBV-associated HCC, HOTAIR is overexpressed in tumor tissues from HCC patients and in liver cancer cell lines, and is related with poor prognosis of HCC, HBx upregulates lncRNAHULC by inhibiting P18 and promoting the occurrence of HCC (103, 105, 107, 108). Additionally, the up-regulated expressions of lncRNA-HEIH and HOTTIP promoted tumor progression and significantly associated with tumor progression and disease outcome in HCC patients (109, 110). Low expression of lncRNA-MEG3 was observed in HCC tissues and cells, and overexpression of lncRNA-MEG3 could inhibit the proliferation, migration and invasion of HCC cells (111). LncRNA Low Expression in Tumor (lncRNA-LET) and lncRNA-SRHC were generally downregulated in HCC, which was associated with hepatic invasion and abdominal metastases (112, 113). Exosomal miR-21 can inhibit the expression of the lncRNA-PTENp1 to promote HCC growth, miR-21 inhibitors or lncRNA-PTENp1 overexpression can weaken the role of exosomal miR-21, which indicates that PTENp1 can repress the tumorigenic properties of HCC cells (114). These findings indicate that lncRNAs play critical regulatory roles in the proliferation, migration and invasion of HCC cells.

### CircRNAs

CircRNAs (circular RNAs), is a new type of non-coding RNA with a closed circular structure without a 5′-end cap and a 3′-end poly A tail. Most of circRNAs are formed by exon loops encoding polypeptides, but some are lariat structures formed by intron loops without encoding ability. Currently, the biological functions of circRNAs are recognized as miRNA sponges, regulatory protein binding, regulation of gene transcription, and coding functions (126). Currently, the circRNAs in human body fluid have been identified in human disease including cancers, autoimmune diseases and infectious diseases. For example, circ-KIAA1244 serves as a novel circulating biomarker to detect gastric cancer (115). CircRNA_0001178 and circRNA_0000826 are considered potential diagnostic biomarkers for liver metastases from colorectal cancer (117). Differential expression of circRNAs and lncRNAs is found in recurrent COVID-19 patients (118). These reports indicated that circRNAs could serve as biomarkers for the diagnosis and therapeutic intervention of human diseases.

To date, the most common mechanism by which circRNAs act as miRNA sponges and interact with certain mRNAs and miRNAs is *via* competing endogenous RNAs (ceRNAs) (127, 128). RNA sequencing revealed that circRNA cSMARCA5 is downregulated in HCC, inhibiting the growth and migration of hepatocellular carcinoma cells, and is associated with a poor prognosis (97). Furthermore, Wu et al. reported that the combination of circ_0009582, circ_0037120, circ_0140117, and AFP has a high sensitivity and specificity to predict HCC (116). Zhang et al. reported that hsa_circ_0001445 levels in plasma are significantly downregulated, which had high specificity (94.2%) and sensitivity (71.2%) in HCC patients, and the efficient combination of plasma hsa_circ_0001445 and AFP levels can be used for HCC diagnosis rather than each parameter alone (129). Furthermore, another circRNA, hsa_circ_104075, was significantly increased in serum from HCC patients, and the AUC value of hsa_circ_104075 (0.973) suggested high sensitivity of 96.0% and specificity of 98.3% (119). Plasma three circRNAs (circ_0009582, circ_0037120, and circ_0140117) were overexpressed in HCC patient, and the combination of the three circRNAs and AFP acquired both valuable positive predictive value (PPV) and negative predictive value (NPV) of 95%, suggesting that these three circRNAs can predict HBV-associated HCC patients or healthy individuals (116).

In addition to the typical miRNA sponging mechanism, post-transcriptional modification of circRNAs may influence translation. The phenomenon was confirmed by overexpressed circ_KIAA1429 in HCC (120). Some circRNAs are associated with drug resistance in HCC treatment. For PD-1 antibody-mediated immunotherapy, circMET makes HCC cells resistant to PD-1 by enhancing the therapeutic microenvironment of immunosuppressive tumors (130). The mechanism revealed that circMET as a sponge of miR-30-5p could promote SNAIL-mediated dipeptidyl 4 (DPP4) expression, leading to CXCL10 degradation, and CXCL10 is a key chemokine in driving intratumor infiltration of effector T cells and may cause subsequent resistance to anti-PD-1 therapy.

Currently, exosomal circRNAs play significant roles in HCC progression, and several exosomal circRNAs function as either diagnostic/prognostic biomarkers or oncogenic/tumor-suppressive factors in HCC (65, 131). The first study in 2015 demonstrated the presence of abundant circRNA inexosomes (132). Previous studies have shown that three exosomes secreted by HCC cells with high metastatic potential, the circPTGR1 subtype, can enhance HCC metastasis with low metastatic potential *via* the miR449a-Met signaling pathway (133). The exosomal circ-0051443 is produced from normal cells and transferred into HCC cells to inhibit the progression of HCC through competitive combination with miR-331-3p and aggravating apoptosis and cell cycle arrest of HCC cells (134). Therefore, recently described properties of circRNAs can not only help us improve understanding but also contribute to the clinical diagnosis and treatment of HCC.

## Proteomics Characteristics

Proteomics are a large-scale study to unveil the profile of proteins expressed under certain biological conditions (135). Recently, proteomics has been used to analyze the overall level of proteins to investigate the pathogenesis, cellular patterns, and functional practices of HBV-associated HCC. With alterations in protein expression in the progression of HCC, some proteins can be considered as potential biomarkers for diagnosis and therapy (136). In a validation study, 28 proteins could separate acute-on-chronic liver failure (ACLF) from CHB patients, the proteomic features developed in this study reflected deficiencies of important hematologic functions in patients with HBV-ACLF, and demonstrated the potential for diagnosis and risk prediction of HBV-ACLF, complementing current clinical-based parameters (137). Compared with serum sample, urine sample is non-invasive and easy to collect, making it more suitable for HCC surveillance in high-risk patients who require frequent examination. Seven protein features were selected in a previous study; among them, HPX, APOH, APCS and PLG were upregulated in HCC urine samples, and GOT1, GLRX, and NCR3LG1 were downregulated (138).

Presently, mass spectrometry is considered a means of protein identification. Pollination mass spectrometry has developed rapidly with the emergence of electrospray ionization mass spectrometry (ESI) and matrix-assisted laser desorption and ionization time of flight mass spectrometry (MALDI-TOF-MS), which provide technical support for proteomics research. Because of its high throughput and sensitivity, MALDI-TOF-MS has provided an optimal response surface for proteomics research as an advanced technique in recent years. The application of MALDI-TOF-MS by the translocation of boron effectively detects the differential serum proteins of HBV-associated HCC, providing important support for diagnosis and treatment of HBV-associated HCC. Tandem mass tag (TMT), isobaric tags for relative and absolute quantification (iTRAQ), stable isotope labeling by amino acids in cell culture (SILAC), and liquid mass spectrometry are used to identify differential proteins. Among them, iTRAQ is considered as one of the most robust quantitative proteomics techniques (139). Compared with 2D gel electrophoresis, iTRAQ technology has many advantages including recognition of low-abundance proteins and high-throughput capabilities. Based on iTRAQ quantitative comparative proteomics, researchers have utilized liquid chromatograph-mass spectrometer/mass spectrometer (LC-MS/MS) to recognize and quantitate differential proteins in HepG2 cell lines stably containing different functional domains of HBx, and p90 ribosomal S6 kinase 2 (RSK2) has been identified as a new host protein that plays a key role in HBx enhancing HBV replication (140). Plasma fibronectin was demonstrated to be related with serum clearance of HBsAg and may be a potential predictor of “functional cure” of CHB by iTRAQ-based quantitative proteomics (141), meanwhile the TMT isobaric labeling-based technology was used to quantitatively characterize the renal proteome of HBV transgenic mice, and to elucidate the pathogenesis of HBV-associated glomerulonephritis (HBV-GN) (142). Additionally, proteomic analyses of formalin-fixed paraffin-embedded (FFPE) HCC graft samples, conducted using a label-free proteome mass spectrometry workflow, were used to characterize the global quantitative analysis of protein expression profiles after gene therapy and to identify differentially expressed proteins (143). Thus, a proteomic strategy to identify HCC candidate biomarkers requires more integrated analysis, and no single methodology can perform this function.

At the same time, the application of proteomics also plays a role revealing the mechanism of the regulation of HBV viral protein in HCC progression, such as HBx, HBs, and HBc. High expressions of GNA13 and GNAi3, belong to the members of the guanine nucleotide-binding protein subunit α (GNA) protein family, are involved in the development of liver cancer through positive and negative regulatory mechanisms, respectively (144, 145). Additionally, recent studies found that the HBx protein promotes the expression of the DNA methylation enzymes DNMT1 and DNMT3A, thereby increasing the methylation level of CpG islands in the promoter region of GNA14 and inhibiting GNA14 expression (146). Endoplasmic reticulum (ER) dysfunction is closely associated with malignant transformation, particularly liver transformation (147). Reticulon (RTN), which is located in the ER, is important for ER maintenance (148). Relevant research results show that HBsAg promotes HCC development by inducing non-mutagenic inactivation of the p53 signaling pathway through the interacting protein RTN3, and proteomic analysis of HBV core protein (HBc) interactions in the nucleus of HepaRG cells revealed that the interaction of HBc with multiple RNA-binding proteins (RBPs) that regulate viral mRNA metabolism provides a new perspective to develop novel host-targeted antiviral strategies (149). Ribosome profiling (RiboSeq) is a novel technology, which could accurately locate the position of ribosomes on mRNA. By combining with RNA-ribosome profiling and proteomics, novel post-translational events hereby detected were then characterize. One study integrated multi-omics analysis, such as RNA-seq, Ribosome profiling and quantitative mass spectrometry, uncovered that an RNA element derived from HBV enhancer I forms a stem-loop which suppresses HBV translation (150). Furthermore, 11 RBPs (RAN, BRIX1, SMG5, DYNC1H1, PRKDC, GTPBP4, and so on) are associated with the overall survival of HCC patients by integrating RNA sequencing and proteomic data (13). These RBPs bind to various RNAs, such as mRNAs, rRNAs, ncRNAs, play a critical role in post-transcriptional gene regulation (PTGR) and are associated with RNA splicing, transport, maturation, degradation, stability, and translation (151). Therefore, they may be drug targets that will help optimize future clinical therapies.

The role of post-translational modifications (PTMs) includes modification events of biochemical functional groups, such as phosphorylation, glycosylation, ubiquitination and so on, which also play an key role on regulating development of HCC. Studies have proved that PTMs are very rich, and the same protein may be modified at multiple sites, which contributes to the diversity of protein structure and function (152). Recent studies have shown that HBV proteins can be modified by different types of PTMs, which affect their protein-protein interaction, subcellular localization and function (153). Recently, multi-omics platforms have performed to systematically interrogate HBV-host interactions. At the transcriptome, proteome and phosphoproteome levels of liver cancer tissues, it was observed that the key enzymes of glycolysis pathway (HK2, ALDOA, PKM2) were significantly up-regulated, indicating that liver cancer has an increased demand for glucose metabolism, and phosphorylation of glycolytic enzymes including ALDOA, may drive metabolic reprogramming and proliferation in liver cancer with CTNNB1 mutation (14). A proteomic analysis had identified that SRSF10as a RNA-binding proteins (RBPs) could be able to alter its phosphorylation and then to regulate HBV RNA metabolism (154). Using high-resolution mass spectrometry, 22,539 phosphorylation sites on 5431 proteins had identified in an HBx-transgenic mouse model of HCC, and these phosphoproteome data highlight potential mechanisms of kinase regulation, especially kinase activities of Src family kinases (SFKs), PKCs, MAPKs, and ROCK2 in HCC (155). Hu et al. revealed the relationship between metabolic reprogramming and antiviral innate immunity against HBV infection usingLC-MS/MS. And O-linked-N-acetylglucosaminylation (O-GlcNAcylation) was proved to regulate host antiviral response against HBV, and O-GlcNAcylation of SAMHD1, as an effector of innate immunity, could stabilize samhd1 structure and enhance host antiviral activity (156). Protein glycosylation is a well-known post-translational modifications and analysis of which based on MS technology commonly. It was reported that the change of glycan heterogeneity in HCC promotes the occurrence, progression and metastasis of tumor, and N-glycosylation is related to the development and progression of HBV-related HCC (157, 158). They showed that altered N-glycopeptide may be part of the unique glycan characteristics, indicating the IgA mediated mechanism and providing potential diagnostic clues for HBV-related HCC. Interferon-α (IFN-α) signaling is crucial for antiviral response. Through high-throughput RNAi screening, Chen et al. identified that the methylation of STAT1 catalyzed by methyltransferase SETD2 was determined to be IFNα-dependent antiviral immunity and showed the potential of SETD2 in controlling HBV infection (159). In the process of viral infection, ubiquitin system is an important part of cellular defense mechanism. Recent studies have shown that ubiquitination may be involved in the degradation of host protein after HBV integration, and there is a negative correlation between the whole proteome and ubiquitin group by performing an Ubiscan quantifcation analysis based on stable isotope labeling of amino acids in cell culture (SILAC) of HepG2.2.15 and HepG2 cell lines (145). Overall, HBV infection mediated changes in post-translational modifications will provide valuable data for further study of the pathogenesis of HBV-related HCC.

## Metabolomics Characteristics

The liver is an internal organ in the human body and is responsible for substrate metabolic and detoxification activities. As a hepatotropic virus, the infectious status of HBV affects liver metabolic function. Understanding how HBV infection relates to hepatic metabolism may provide new insights into the pathogenesis of HBV infection.

Metabolomics is the study of the profile of metabolites (e.g., amino acids, lipids, sugars, and hormones) that are detectable under certain conditions. Tumors from HCC patients may alter metabolic pathways, and the resulting changes in nutritional supply are essential to overcome nutritional hunger and changes in environmental conditions (160). Compared with other “omics,” metabolomics not only provides the most direct snapshot of the actual functional and physiological state of biological networks but also establishes a key technique to investigate metabolic alterations in carcinogenesis (161, 162). Currently, no standard or routine screening test exists for liver cancer. X-ray computed tomography scan, ultrasound and α-fetoprotein (AFP) are the typical tests used to screen for liver cancer, while liver biopsy is used as the gold standard (163). Metabolomics studies uncover new insights into the biological understanding of HCC and reveal particular implications related to clinical and therapeutic plans. The main techniques applied to metabolomics are nuclear magnetic resonance spectroscopy (NMR), gas chromatography-mass spectrometry (GC-MS) and LC-MS (164). Recent studies based on mass spectrometry and next-generation sequencing unveiled the active status of signaling pathways and reprogramming of hepatic metabolism in HBV-associated HCC at the genomic and proteomic levels (14, 38). MS-based technologies can provide measures of the global changes in protein abundance related to the deregulation of signaling and metabolic pathways in HCC. NMR spectroscopy-based metabolomics provide a non-targeted, quantitative snapshot of global metabolite abundance to provide additional biological insights that cannot be deciphered by proteomics alone (165). Additionally, the combination of GC-MS- and NMR-based metabolomic platforms is promising because the application of multi-metabolomics platforms yields a superior biomarker panel to diagnose bipolar disorder (166). Previously, in the field of metabonomics, substantial efforts have been made to search for biomarkers of HCC, some of which are candidate biomarkers (167). However, how the metabolic phenotype is driven remains unclear in HBV-associated HCC.

The metabolomics profile identified in HCC offers unprecedented opportunities to screen candidate metabolites for early diagnosis and treatment. From the perspective of metabolomics, lipid, energy and amino acid metabolism may be affected in the progress of HCC (168). Glycolysis-related metabolites, TCA cycles and pyrimidine synthesis change in tumor tissues at different stages. Carbohydrates that are energy sources of hepatocytes and carbohydrates, such as mannose, galactose, and arabinose, are significantly reduced in the serum of HCC patients and other liver diseases (169). The reductional feature of carbohydrates in HCC is also consistent with most cancer cells, in which they can produce energy by undergoing high speed glycolysis followed by lactic acid fermentation in the cytoplasm instead of using oxidative phosphorylation in mitochondria (170). Dysregulation of amino acid metabolism is associated with liver disease and HCC development (171). Because of increased tumor protein synthesis and energy demand of amino acids in malignant tumor cells (172), multiple amino acids, such as proline, lysine, ornithine, phenylalanine serine, and tyrosine, are upregulated significantly in HCC and HBV-cirrhosis patients (165). Additionally, because of aggressive cell proliferation in HCC, the energy supply and cell membrane synthesis must increase fatty acids including arachidonic acid, which is at a higher level in HBV-cirrhosis and HCC patients (173, 174). Thus, the fatty acids may be involved in the pathogenesis of HCC.

During the viral life cycle, HBV is associated with hepatic metabolism. This evidence of the involvement of cell metabolism in HBV-associated cancer prognosis raises interest in metabolic enzymes-targeted cancer therapy (175). In the study of the metabolic pathway, omics evidence also shows that immune patterns between HBV and the host are closely associated with the disease progression of patients infected with viruses, and metabolic alterations can be regulated by HBV protein in HCC cells. Through multiomic analysis, Xie et al. demonstrated that HBV core protein (HBc) increased the secretion of metabolites and expression of metabolic enzymes in HCC cells, and activated the amino acid and glycolysis metabolism pathways (176). Similar to previous studies, HBc can bind to human gene promoters to mediate primary metabolic processes (177). The metabolic components of liver microenvironment are actively involved in the occurrence and development of HBV infection, and hot-spot mutations in HBc, including L60V, I97L, and S87G, affect viral replication, persistence and immune pathogenesis in CHB infection (178, 179). Additionally, HBs, HBx, and HBc integrate into human genes to affect patient survival (47). Cellular retinoid X receptor alpha (RXRα), a key transcription factor for monitoring hepatic lipid metabolism, regulates HBV infection, and the arachidonic acid (AA)/eicosanoid biosynthesis pathway may be involved in the regulation of HBV infection (180). Therefore, hepatic lipid homeostasis is critical to modulate viral infection.

## Microbiome Characteristics

Microbiome is becoming a potentially key regulator of cancer development, especially in gut and liver microbiomes. Because the microbial group is mainly located in the intestines, the gut microbiome is the most studied and associated with a variety of human diseases, including Alzheimer's disease, cardiovascular disease, diabetes, arthritis and cancer, which is not surprising (181). Actually, gut bacteria play a key role in maintaining gut-liver axis health, and intestinal flora disorders occurred in 20–75% of patients with chronic liver disease (182). One study carried out 16S rRNA analyses in 35 individuals with HBV related HCC (B-HCC). Compared with 22 individuals with non-HBV/non-HCV (NBNC) related HCC (NBNC-HCC), the species richness of fecal microbiota of B-HCC patients was much higher. The results showed that there are differences in the number of bacteria involved in different functions or biological pathways (183). Zheng et al. also showed that gut microbiota disorder was more common in patients with liver cirrhosis-induced HCC, however, hepatitis virus infection was not associated with intestinal microbial imbalance. The data indicated that butyrate-producing genera was decreased and genera producing-lipopolysaccharide (LPS) was increased in liver cirrhosis-induced HCC (184). In HBV induced tumors, this tumor inhibitory effect is inferred based on the down-regulation of microorganisms that induce cancer and stem cell pathway. Using next-generation RNA-sequencing against HBV-related HCC patients and adjacent normal liver tissues, the results of this study suggest that both heavy drinking and HBV infection may use the tumor microbiome to promote the development of cancer, however, only HBV infection could downregulate microorganisms that may promote stem cell function (185). They suggested strains of *Escherichia coli* were to be potentially important to HCC progression. The change of liver microenvironment in HCC patients may lead to the change of bacterial level in gut. Overall, gut-liver-axis could be used to monitor and prevent the progress of liver disease and liver cancer.

## Conclusions

The initiation and progression of liver cancer are involved in multisystem and multilevel pathological changes. Single-omics analysis plays a key role in the diagnosis and therapy of diseases in modern society. However, with the development of research technologies and needs, single-omics is not sufficiently comprehensive; one type of omics change can't represent the overall status of the disease, only 10–20% of transcriptome changes are associated with proteomic data (186). Abnormal gene expression is also a risk factor leading to tumor cell formation. Changes in DNA nucleotide sequences and epigenetic mechanisms may result in aberrant gene expression profiles. The entire regulatory network may be clearly illustrated by more advanced and sensitive high-throughput omics technologies. Single-omics research is crucial, and this method often has some limitations. By contrast, the integrated analysis of multi-omics data can better describe the overall changes in liver cancer, thus achieving more valuable data in the diagnosis and development of therapeutic targets in human diseases. The latest advances in analytical technology, including ultra deep sequencing, have made multi-omicsmulti-omics analysis faster, more accurate and simpler. Many omics technologies are widely used in cancer research. The introduction of omics technology to analyze the pathogenesis or treatment of HBV-associated HCC may help not only to identify biomarkers for clinical use but also to explore the experimental research background of the pathogenesis of various diseases. Several studies have detected exome sequencing or whole genome sequencing and have used genome sequencing to identify driver gene mutations in liver cancer. The application of currently rapidly developing omics technology will promote the development of knowledge-based diagnosis and treatment strategies. In the future, more cohort studies will explore the prognostic factors of HCC patients, and candidate genes related to prognosis or recurrence of HCC will be identified using omics technology.

## Author Contributions

YW, DC, and JX conceived the topic. YW and DC conducted literature review, drafted the manuscript, designed the figures, and tables. JX, WL, JW, and DJ polished the manuscript. All authors approved the submitted version.

## Funding

This work was supported by the Zhejiang Provincial Natural Science Foundation of China (No. LBY21H190001), National Natural Science Foundation of China (Nos. 81871709, 91846103, and 81971994), and Zhejiang Provincial Key Research and Development Program (No. 2020C03032).

## Conflict of Interest

The authors declare that the research was conducted in the absence of any commercial or financial relationships that could be construed as a potential conflict of interest.

## Publisher's Note

All claims expressed in this article are solely those of the authors and do not necessarily represent those of their affiliated organizations, or those of the publisher, the editors and the reviewers. Any product that may be evaluated in this article, or claim that may be made by its manufacturer, is not guaranteed or endorsed by the publisher.
